# Anaerobic Digestion of the Liquid Fraction of Fruit and Vegetable Waste: Two-Stage versus Single-Stage Process

**DOI:** 10.1021/acsomega.5c00073

**Published:** 2025-05-29

**Authors:** Francisco das Chagas Gomes da Silva Júnior, Priscilla de Souza Almeida, Camila Aparecida de Menezes, Maíra Saldanha Duarte, Thobias Pereira Silva, André Bezerra dos Santos, Marcelo Zaiat, Renato Carrhá Leitão

**Affiliations:** † Escola de Engenharia de São Carlos, 67817 Universidade de São Paulo, Rua João Dagnone 1100, São Carlos, Sao Paulo 13563-120, Brazil; ‡ Embrapa Tropical Agroindustry, Biomass Technology Laboratory, Rua Dra. Sara Mesquita 2270, Fortaleza, Ceara 60511-075, Brazil; § Department of Hydraulic and Environmental Engineering, Federal University of Ceara, Campus do Pici, Bl 710, Fortaleza, Ceara 60440-900, Brazil

## Abstract

This study evaluated the anaerobic digestion of the liquid fraction of fruit and vegetable waste to optimize energy recovery through sequential hydrogen and methane production. Two configurations were tested: a single-stage (SS) system using an upflow anaerobic sludge blanket (UASB) reactor and a two-stage (TS) system combining an anaerobic structured bed reactor (AnStBR) with a UASB reactor. The objective was to identify the most efficient configuration under various organic loading rates (OLR). In the AnStBR, OLR ranged from 40 to 80 g COD/L_reactor_·day, while in UASB reactors, the OLR range was from 1.5 to 22.0 g COD/L_reactor_·day. The TS outperformed the SS in methane production, achieving 3.6 NL CH_4_/L_reactor_·day at an OLR of 22.0 g of COD/L_reactor_·day. It also demonstrated a higher energy potential, generating a total of 1554.9 KJ/day—35.7% more efficient than the SS. Additionally, the UASB-TS reactor maintained stability throughout the study, with minimal acid accumulation and an intermediate alkalinity/partial alkalinity ratio (IA:PA) consistently below 0.4, even at elevated OLR. In contrast, the UASB-SS reactor showed signs of acidification at higher OLR. These findings suggest that TS anaerobic digestion offers a more robust and efficient solution for treating high-strength organic waste, enhancing both energy recovery and process stability.

## Graphical abstract

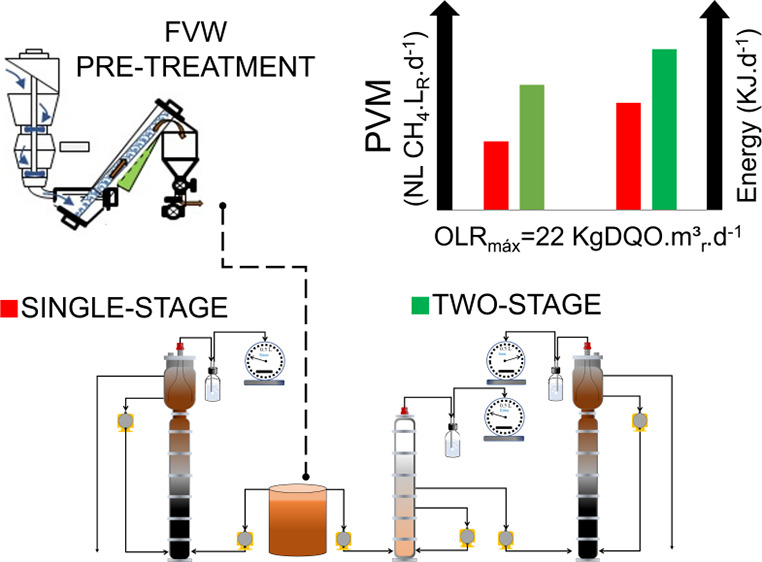

## 1 Introduction

In recent years, solid waste management has emerged as a pivotal topic in global discussions on environmental sustainability. A key objective within the United Nations’ Sustainable Development Goals (SDG) is to reduce food loss and waste by 50% by 2030.^1^ Despite this, projections estimate that global food waste could reach approximately 2.6 billion tons by that time.^2^ Current data indicate that around 1.3 billion tons of food are wasted annually worldwide, representing nearly one-third of all food produced for human consumption.^3^

This magnitude of waste not only exacerbates global food insecurity but also significantly depletes natural resources, such as land, water, and energy. Furthermore, food loss and waste account for approximately 8% of global greenhouse gas emissions, thereby intensifying environmental challenges.^4^ The agro-industrial sector, particularly the food processing industry, stands out as one of the largest producers of biodegradable solid waste.^5,6^ Among the various waste management strategies, anaerobic digestion of fruit and vegetable waste (FVW) has demonstrated significant potential for biogas production.^7,8^

FVW, rich in soluble carbohydrates such as glucose, fructose, and sucrose, serves as an excellent carbon source for anaerobic digestion, enhancing bacterial activity and facilitating biogas production.^9^ Anaerobic digestion presents a promising approach for FVW treatment, yielding renewable energy in the form of biogas—primarily methane (CH_4_) and hydrogen (H_2_)—along with biofertilizers.^10,11^ However, optimizing organic loading rate (OLR) and developing strategies to mitigate volatile fatty acid (VFA) accumulation are critical for improving the efficiency and sustainability of biogas production.^12^

In single-stage (SS) reactors, the rapid degradation of FVW often results in the accumulation of VFA and excessive acidification.^13^ This instability restricts the application of higher OLR, as methanogenic archaea are inhibited at low pH levels, potentially leading to reactor failure.^14,15^ To mitigate these challenges, two-stage (TS) anaerobic digestion systems separate the acidogenic and methanogenic phases, enabling more precise control over critical parameters such as the pH and hydraulic retention time (HRT). This approach promotes operational stability and enhances energy recovery efficiency.^16^

TS digestion of FVW not only facilitates the application of higher OLR in anaerobic reactors but also achieves methane yields (MY) up to 35% greater than those observed in SS processes.^17^ Almeida et al.^17^ reported a maximum OLR of 1.5 g COD/L_reactor_·day with an HRT of 20 day for FVW digestion in a TS continuously stirred tank reactor (CSTR). In comparison, a CSTR-SS—the conventional configuration for this type of residue—attained an OLR of only 1.0 g of COD/L_reactor_·day with an HRT of 34 day.

A review by Dangol et al.^18^ on TS anaerobic digestion of food waste highlighted that the HRT in the hydrogenogenic first-stage reactor typically ranges from 1 to 3 day, while the methanogenic second-stage reactor operates with an HRT from 10 to 15 day. In this study, strategies such as separating solid and liquid fractions, milling followed by centrifugation, and using high-rate reactors are expected to reduce the HRT and increase the OLR in SS and TS reactor configurations, ultimately enhancing the performance. Notably, the dark fermentation of the liquid fraction of FVW (FVWL) in a high-rate reactor, specifically the Anaerobic Structured Bed Reactor (AnStBR), enabled the application of an HRT of just 6 h^19^—significantly lower than the 1 to 3 day range reported in the literature. Additionally, in the SS reactor, Upflow Anaerobic Sludge Blanket (UASB), an HRT of 1 day was applied,^20^ also lower than the values typically found by Dangol.^18^

Although TS digestion shows significant promise, it does not fully address the challenges posed by the high total solids content of FVW, which averages around 18%.^17^ Separating the FVWL from the solid fraction may enhance the efficiency of anaerobic digestion processes.^19^ In this study, an AnStBR and an UASB reactor were utilized—a configuration that supports biomass retention and microbial stability in the acidogenic phase (AnStBR) at a short HRT of 6 h^19^ and enables high-rate effluent treatment during the methanogenic phase (UASB) with an HRT of 24 h.^10^ This study examined the effect of increasing the OLR on H_2_ and CH_4_ production in the TS anaerobic digestion of FVWL using AnStBR and UASB reactors. The performance of this TS system was compared to that of a SS digestion setup.

## 2 Experimental Methods

### 2.1 Feed Substrate

A monthly quantity of 150 kg of FVW was collected from the Wholesale Supply Center (CEASA) in Maracanaú, Ceará, Brazil. The waste composition, by mass percentage, was as follows: orange (47.2%), onion (8.6%), corn (6.1%), papaya (6.2%), avocado (5.3%), watermelon (3.8%), melon (3.5%), pineapple (3.5%), banana (3.3%), potato (3.4%), cabbage (2.6%), guava (1.3%), tomato (1.1%), pepper (1.1%), beetroot (1.0%), apple (0.7%), passion fruit (0.5%), carrot (0.4%), and pumpkin (0.2%).

After the standard formulation of the FVW, this waste underwent preliminary mechanical treatment, consisting of mechanized shredding using a forage harvester, generating the shredded waste, followed by basket centrifugation at 150 rpm for 5 min. This process separated the solid (FVWS) and FVWL, representing approximately 55 and 45% of the shredded residue, respectively. The primary distinction between the FVWL utilized in the present study and that in the study conducted by de Menezes et al.^20^ and Cavalcante et al.^10^ lies in the preparation process. In the present study, FVWL was derived from FVW subjected solely to centrifugation. In contrast, Cavalcante et al.^10^ employed FVWL obtained from FVW that underwent both centrifugation and pressing, resulting in a higher concentration of total solids.

For the physicochemical characterization, the FVWL samples were diluted (50 g/L) and homogenized at 10,000 rpm for 15 min using the T25 digital Ultra-Turrax homogenizer (IKA) to reduce errors due to the heterogeneity of the waste. Therefore, the samples were characterized for chemical oxygen demand (COD), total solids, volatile solids (VS), and total Kjeldahl nitrogen according to APHA, AWWA, and WEF.^21^ Total carbohydrates were determined according to Dubois et al.^22^ The protein content was determined by combustion, using the DUMAS method on a Nitrogen/Protein Analyzer NDA 701 Dumas with EDTA as the standard, based on the AOAC 992.23 method. Lipid concentrations were measured according to the Am 5-04 method of the American Oil Chemists’ Society, using a high-pressure and high-temperature extraction system in an XT-15 Ankom device (ANKON Technology Corporation).

### 2.2 Reactors Setup

The first system consisted of an SS configuration (Figure 1A), comprising an UASB reactor (UASB-SS) with a volume of 12.6 L, designed for CH_4_ production. The second system was a TS setup (Figure 1B), which included an AnStBR as a first stage with a volume of 3.0 L, targeting H_2_ and VFA production. The acidified effluent from AnStBR was subsequently directed to a second UASB reactor (UASB-TS) for sequential CH_4_ production.

**Figure 1:**
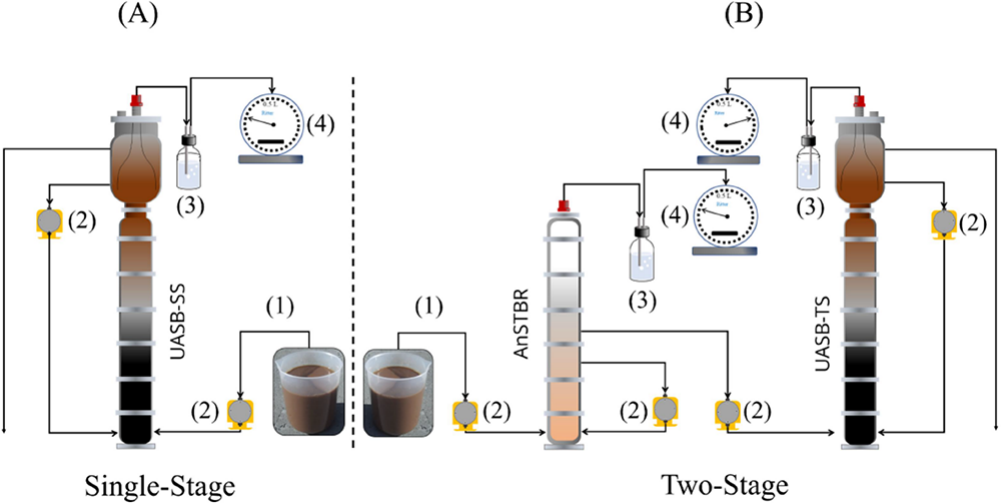
Schematic representation of the two systems: (A) SS system composed of an upflow anaerobic sludge blanket (UASB) reactor and (B) TS system, consisting of a anaerobic structured-bed reactor (AnStBR) followed by an upflow anaerobic sludge blanket (UASB) reactor: (1) FVWL; (2) Pump; (3) Hydraulic seal; (4) Gasometer.

Each UASB reactor was inoculated with 7.0 kg of mesophilic anaerobic sludge obtained from a UASB reactor treating a brewery effluent. The inoculum sludge contained 3.9% of total solids and 68.4% of VS/total solids. For the AnStBR, the inoculum sludge underwent thermal pretreatment at 90 °C with agitation for 10 min, followed by thermal shock as described by Maintinguer et al.^23^ to inhibit methanogenic archaea activity.

The feed flow rate to the reactors was set at 0.5 L/h, resulting in HRT of 6 h for the AnStBR^19^ reactor and 24 h for each of the UASB reactors. In the TS process, the AnStBR was fed with substrate concentrations increased from 10 to 12, 15, 18, and 20 g/L, resulting in an OLR varying from 40.0 to 80.0 g of COD/L_reactor_·day. Meanwhile, the UASB-TS received the effluent from the AnStBR, with nearly the same concentration, leading to an OLR ranging from 1.5 to 22.0 g of COD/L_reactor_·day.

Similarly, in the SS process, the UASB-SS was operated with an OLR comparable to that of the UASB-TS (1.5–22.0 g of COD/L_reactor_·day). The initial OLR applied to both UASB reactors, ranging from 1.5 to 10 kg COD/m^3^·day, enable a direct comparison with the UASB reactor operated by Cavalcante et al.^10^ Each OLR was maintained in UASB-SS and UASB-TS until stabilization, defined as a variation of less than 10% in organic matter removal efficiency and methane production yield over ten consecutive days.

The pH in the AnStBR was maintained between 4.5 and 5.5, and in the UASB reactors, it was kept at 8.5. To ensure pH buffering in the methanogenic reactors, sodium bicarbonate (NaHCO_3_) was added to the feed at a ratio of 0.3–1.0 g NaHCO_3_/g COD_add_.^24^ Both systems were operated under a mesophilic temperature (32 °C).

### 2.3 Sampling and Analysis

The concentration of total solids, VS, COD, Total Kjeldah Nitrogen (TKN), and pH were analyzed according to Standard Methods.^21^ The carbohydrate content, with sucrose as a reference, followed the methodology proposed by Dubois et al.^22^ Total alkalinity and total volatile acids were determined according to Ripley et al.^25^ and Dilallo and Albertson,^26^ respectively.

The volume of biogas generated by the reactors was monitored using a Ritter TG 0.5 gasometer (RITTER Apparatebau GmbH & Co. KG, Bochum, Germany). Gas composition analysis was conducted by gas chromatography (Shimadzu Nexis GC-2030), and the acids were identified by high-performance liquid chromatography (Shimadzu UFLC HPLC System).

The statistical analysis of the data was performed using SPSS software, version 13.0. The statistical parameters obtained were the mean, variance, and standard deviation. The paired *t*-Student method and analysis of variance (ANOVA) were used to determine statistical significance (*p*-value ≤ 0.05).

## 3 Results and Discussion

### 3.1 Characterization of Substrates

Table 1 shows the main characterization results of FVWL, including total COD and VS, and a comparison with similar substrates reported in the literature. The pH of the residue was 4.5 ± 0.1, with acidic characteristics attributed to the high proportion of oranges (47.2%) in the FVW composition. In general, FVW have low pH (<6.0), which promotes acidification and consequently an accelerated production of VFA, potentially limiting anaerobic digestion for this type of substrate.^27^ The characterization of FVWL aligns with literature values for potential substrates for anaerobic digestion.

**Table 1:** Characterization of FVWL Obtained at CEASA-CE and Comparison with the Literature^a^

parameter	this study	de Menezes et al.^20^	Cavalcante et al.^10^	Martínez-Mendoza et al.^28^
total COD (g COD/kg_substrate_, w.w.)	115.8 ± 4.2	116.0	137.1	111.5
total solids (%,w.w.)	8.2 ± 0.3	11.0	11.0	9.5
moisture content (%)	90.8 ± 0.3	90.6	91.7	90.5
VS (%total solids, w.w.)	93.1 ± 0.1	93.6	90.1	94.0
COD/VS ratio (kg COD/kg VS, d.w.)	1.24 ± 0.2	1.33	1.7	1.24
total carbohydrate (g/L, w.w.)	45.6 ± 2.9	45.7	55.2	78.9
lipids (% d.w.)	0.7 ± 0.1	0.8	0.2	1.2
raw proteins (%w.w.)	1.1 ± 0.1	1.2	1.7	15.5
total nitrogen (g/kg_substrate_, d.w.)	15.2 ± 0.1	15.0	n.d.	n.d.
C/N (g COD/g TN, d.w.)	7.61	23.8	n.d.	31.6
pH	4.5 ± 0.1	n.d.	4.4	4.6
acetic acid (mg/L, w.w.)	176.7 ± 36.2	162.0	n.d.	n.d.
lactic acid (mg/L, w.w.)	258.2 ± 128.9	n.d.	n.d.	n.d.
isobutyric acid (mg/L, w.w.)	505.1 ± 150.1	192.0	n.d.	n.d.

a Values ± standard deviation. w.w. = wet weight. d.w. = dry weight. n.d. = not determined.

Although the FVW used in this study originated from the same initial composition as that in the works of de Menezes et al.^20^ and Cavalcante et al.,^10^ differences in pretreatment processes—specifically the pressing of the residue—led to variations in total solids concentration. The higher total solids content in the SS UASB reactor operated by Cavalcante et al.^10^ resulted in challenges such as sludge washout and solid accumulation within the reactor. These issues constrained the OLR to a maximum of 10 g of COD/L_reactor_·day. In contrast, reducing the total solids concentration, as done in the present study, is expected to enable the application of the OLR exceeding 10 g of COD/L_reactor_·day, even in SS UASB reactors, thereby improving process efficiency and performance.

FVW generally contain adequate concentrations of potassium (K), calcium (Ca), and magnesium (Mg) but show deficiencies in cobalt (Co), nickel (Ni), selenium (Se), and molybdenum (Mo).^29,30^ These elements are essential for microbial growth and efficient biogas production during anaerobic digestion. Table 2 presents the main results of the mineral characterization of the FVWL used in this study.

**Table 2:** Mineral Characterization of FVWL^a^

minerals	unit	values ± s.d.
total phosphorus (P)	(g/kg d.w.)	3.0 ± 0.1
potassium (K)	(g/kg d.w.)	25.7 ± 0.3
calcium (Ca)	(g/kg d.w.)	2.5 ± 0.0
magnesium (Mg)	(g/kg d.w.)	1.7 ± 0.0
sulfur (S)	(g/kg d.w.)	1.2 ± 0.1
sodium (Na)	(g/kg d.w.)	0.8 ± 0.0
copper (Cu)	(g/kg d.w.)	4.8 ± 1.0
iron (Fe)	(g/kg d.w.)	406.2 ± 4.1
zinc (Zn)	(g/kg d.w.)	13.9 ± 1.4
manganese (Mn)	(g/kg d.w.)	19.4 ± 0.6

a s.d.: standard deviation. d.w. = dry weight.

However, nutrient composition is often overlooked, based on the assumption that trace elements are naturally present in the various waste components.^31^ Nevertheless, ignoring deficiencies in nutrients such as nickel, zinc, and iron can delay the growth of methanogenic microorganisms, resulting in toxicity and even complete inhibition of CH_4_ synthesis.^32,33^ Cobalt, for instance, is uncommon in vegetable waste and thus must be supplemented, as it is essential for the synthesis of vitamin B12, required for microbial metabolism.^34^ Sulfur, on the other hand, present in waste containing sulfur-rich foods like onion, avocado, guava, and corn, can increase dissolved H_2_S production, becoming toxic to anaerobic microorganisms.^35^ Therefore, to guarantee the necessary elements for efficient metabolic activity of the microorganisms, the reactor feed was supplemented with micro- and macronutrients.^36^

### 3.2 Influence of OLR on the Performance of the Hydrogenogenic Reactor

The AnStBR was operated with an OLR ranging from 40.0 to 80.0 g of COD/L_reactor_·day. The pH was maintained within the range of 4.5–5.5 (Figure 2A), a condition that promotes the production of lactic acid over H_2_ production.^37^ However, some studies suggest that H_2_ can be produced from lactate.^38^ The high production of VFA (Figure 2B) and H_2_ (Figure 2C) observed in the AnStBR at low pH (4.0–5.0) may be attributed to this phenomenon, as H_2_ production from lactate has been documented to occur within a pH range of 3.8–7.5.^39^ Several metabolic pathways for converting lactate to H_2_ have been identified in the literature, involving bacteria of the *Clostridium* genus (eqs 1 and 2) and mixed cultures (eqs 3 and 4).^40^ In these pathways, simultaneous production of butyrate and acetate, alongside H_2_, is observed. According to Brodowski et al., the presence of acetate promotes the utilization of lactate for H_2_ production through an acetate/lactate pathway.^41^

$${\mathrm{CH}}_{3}\mathrm{CH}\left(\mathrm{OH}\right)\mathrm{COOH}\to 0.5{\mathrm{CH}}_{3}{\mathrm{CH}}_{2}{\mathrm{CH}}_{2}\mathrm{COOH}+H_{2}+{\mathrm{CO}}_{2}+0.5H_{2}O$$ 1

$${\mathrm{CH}}_{3}\mathrm{CH}\left(\mathrm{OH}\right)\mathrm{COOH}+H_{2}O\to {\mathrm{CH}}_{3}\mathrm{COOH}+2H_{2}+{\mathrm{CO}}_{2}$$ 2

$${\mathrm{CH}}_{3}\mathrm{CH}\left(\mathrm{OH}\right)\mathrm{COOH}+0.28{\mathrm{CH}}_{3}\mathrm{COOH}\to 0.67{\mathrm{CH}}_{3}{\mathrm{CH}}_{2}{\mathrm{CH}}_{2}\mathrm{COOH}+0.47H_{2}$$ 3

$${\mathrm{CH}}_{3}\mathrm{CH}\left(\mathrm{OH}\right)\mathrm{COOH}+0.5{\mathrm{CH}}_{3}\mathrm{COOH}\to 0.75{\mathrm{CH}}_{3}{\mathrm{CH}}_{2}{\mathrm{CH}}_{2}\mathrm{COOH}+0.5H_{2}+{\mathrm{CO}}_{2}+0.5H_{2}O$$ 4

**Figure 2:**
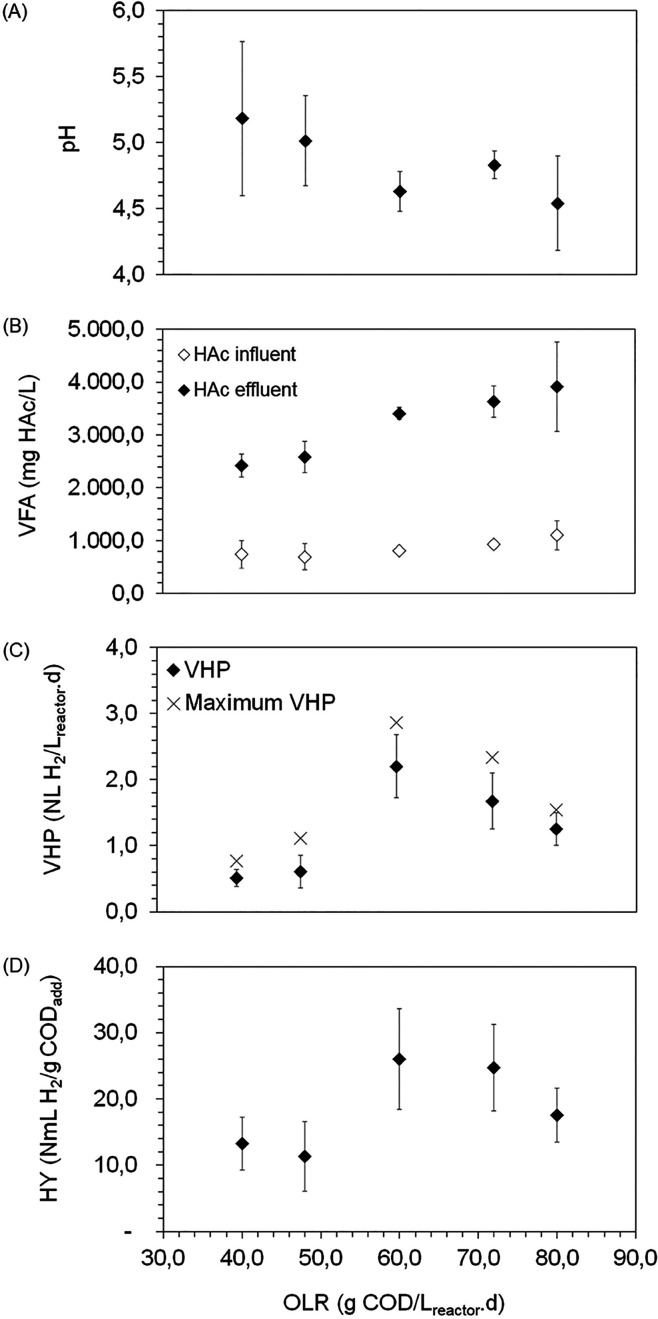
(A) pH, (B) volatile fatty acids, (C) hydrogen production (VHP), and (D) hydrogen yield (HY) under different organic loading rates (OLR) (40–80 g COD/L_reactor_·day) applied to the AnStBR reactor.

Recent studies have demonstrated that the production of H_2_ from FVW via lactate-driven dark fermentation can achieve significant hydrogen yields.^39^ Martínez-Mendoza et al.^42^ reported a H_2_ production of 1897.5 ± 370.4 N mL H_2_/L_reactor_ at pH 5.5 and 3443.1 ± 46.4 N mL H_2_/L_reactor_ at pH 7.0, associating this result with a conversion of lactate to H_2_, butyrate, and acetate, since an increase in butyrate and acetate concentrations was observed along with H_2_ production and low lactate concentrations. Martínez-Mendoza et al.^43^ also highlighted that lactate-driven dark fermentation is prominent to achieve high H_2_ productions from FVW.

The OLR significantly influences volumetric H_2_ production (VHP) in anaerobic reactors. However, excessively high OLR can lead to the accumulation of VFA, resulting in lower hydrogen yields (HY),^44,45^ as observed in Figure 2D. In this study, the variation at the OLR in the AnStBR influenced VHP, ranging from 0.5 ± 0.1 to 1.3 ± 0.3 L H_2_/L_reactor_·day. Nevertheless, the highest average H_2_ production (2.2 L of H_2_/L_reactor_·day) was observed at an OLR of 60.0 g of COD/L_reactor_·day, with peak values reaching 2.9 L of H_2_/L_reactor_·day. For instance, Paudel et al.^46^ reported a maximum H_2_ production of 0.44 L H_2_/L_reactor_·day in the acidogenic CSTR, using a mixture of food waste as the substrate at the OLR of 106.0 g TS/L_reactor_·day. In another study, Araujo et al.^47^ using an AnStBR with the same HRT of 6 h as in this study and fermenting sucrose observed a maximum VHP of 0.7 L H_2_/L_reactor_·day. Using FVW as the substrate, de Menezes et al.^19^ observed the production of 2.094 L H_2_/L_reactor_·day in an AnStBR at 20 g COD/L_reactor_·day (HRT of 6 h), and with an increase in the OLR to 40 g COD/L_reactor_·day (HRT of 3 h), the production decay to 0.516 L H_2_/L_reactor_·day. These results reinforce that the optimal OLR for H_2_ production depends on the reactor configuration, substrate, and the OLR.

During the operation of AnStBR, carbohydrate conversion remained around 90%, while COD reduction was about 15%. The concentration of each metabolite detected in the AnStBR under an OLR of 40–80 g COD/L_reactor_·day is shown in Table 3. At an OLR of 40.0 g COD/L_reactor_·day, where the lowest VHP was recorded, the total concentration of VFA in the effluent was 2423 ± 218 mgHAc/L, with the major metabolites distributed as butyric acid (1094 ± 124 mgHBu/L), propionic acid (746 ± 97 mgHPr/L), acetic acid (451 ± 22 mgHAc/L), valeric acid (153 ± 16 mgHVa/L), and lactic acid (77 ± 2 mgHLa/L). In contrast, at an OLR of 60.0 g of COD/L_reactor_·day, where the VHP was highest, the concentration of VFA in the effluent increased to 3407 ± 117 mgHAc/L, with butyric acid as the main metabolite (2991 ± 489 mgHBu/L), followed by acetic acid (1107 ± 191 mgHAc/L), isobutyric acid (642 ± 107 mgHIsBu/L), lactic acid (148 ± 54 mgHLa/L), and propionic acid (86 ± 16 mgHPr/L).

**Table 3:** Average Concentrations of Acidic Metabolites Produced in the AnStBR Reactor under Organic Loading Rates (OLR) of 40–80 g COD/L_reactor_·day^a^

		OLR (g COD/L_reactor_·day)				
parameter	unit	40	48	60	72	80
pH		5 ± 0.6	5 ± 0.3	5 ± 0.2	5 ± 0.1	5 ± 0.4
VHP	L H_2_/L_reactor_·day	1 ± 0.1	1 ± 0.3	2 ± 0.5	2 ± 0.4	1 ± 0.3
HY	mL H_2_/g COD	13 ± 4	11 ± 5	26 ± 8	25 ± 7	18 ± 4.
VFA	mg/L	2424 ± 218	2581 ± 298	3407 ± 117	3628 ± 297	3910 ± 849
HBu	mg/L	1094 ± 124	1630 ± 443	2921 ± 489	3357 ± 222	1408 ± 528
HAc	mg/L	451 ± 22	456 ± 128	1107 ± 191	703 ± 149	355 ± 82
HIsBu	mg/L	52 ± 34	317 ± 193	642 ± 107	626 ± 74	690 ± 38
HLa	mg/L	77 ± 2	399 ± 195	148 ± 54	260 ± 86	144 ± 35
HPr	mg/L	746 ± 97	73 ± 19	86 ± 16	62 ± 28	N.D
HVa	mg/L	153 ± 16	86 ± 0.1	N.D	190 ± 52	106 ± 11

a Value ± standard deviation; N.D.: not detected; VHP: volumetric hydrogen production; HY: hydrogen yield; VFA: volatile fatty acids; HAc: acetic acid; HLa: lactic acid; HPr: propionic acid; HVa: valeric acid; HIsBu: isobutyric acid; HBu: butyric acid.

At the highest volumetric H_2_ production, the favored metabolic pathways are associated with the production of acetic acid and butyric acid. These pathways are responsible for the highest hypothetical H_2_ production, i.e., 4 mol of H_2_ per mol of glucose (acetate pathway) and 2 mol of H_2_ per mol of glucose (butyrate pathway).^48^ However, butyric acid at higher concentrations and lower pH levels can inhibit H_2_ production, as a greater fraction of this acid remains in its undissociated form (C_4_H_8_O_2_).^49^ According to Nicolaou et al.,^50^ undissociated organic acids can diffuse through the cell membrane of microorganisms and accumulate in either their anionic or cationic forms, significantly impacting cell physiology. The anionic form of organic acids facilitates the influx of undissociated acids into the cell, promoting a balance between the intra- and extracellular environments. This leads to the accumulation of anions. Additionally, an increase in the concentration of undissociated acids results in a decrease in intracellular pH, creating a disparity between intra- and extracellular pH values that can disrupt membrane integrity.

To counteract the pH drop, cells transport H+ ions out of the cell, which requires ATP expenditure and subsequently affects the energy available for cellular activities. The buildup of free protons can be detrimental to the cell’s RNA and DNA, as well as disrupt enzymatic functions.^50^ Each enzyme operates optimally within specific pH ranges, meaning that pH can influence hydrogen production and metabolic pathways. For instance, phosphotransacetylase and lactate dehydrogenase—enzymes involved in acetate and lactate synthesis, respectively—exhibit peak activity at pH 5, with the activity of phosphotransacetylase being inhibited in the presence of butyric acid.^51^ Furthermore, the activity of hydrogenase, an enzyme responsible for hydrogen production, is diminished at low pH levels,^52^ while it has been reported to be most active at pH 5.0–6.5.^53^

For instance, at concentrations of 60 mM of undissociated butyric acid and a pH of 5.0, H_2_ production can be suppressed by over 93% when using glucose as the substrate in a continuous flow reactor.^54^ This suppression highlights the inhibitory effects of high concentrations of undissociated acids on H_2_ production processes.^54^ Despite their inhibitory potential at high concentrations and low pH, controlled levels of butyric and acetic acids can support H_2_ production in fermentative processes.

In comparison, high concentrations of propionic acid are observed in systems with the lowest H_2_ production. This is because the accumulation of propionic acid is related to the H_2_ consumption, according to eq 5.^48^

$$C_{6}H_{12}O_{6}+2H_{2}\to 2{\mathrm{CH}}_{3}{\mathrm{CH}}_{2}\mathrm{COOH}+2H_{2}O$$ 5

Although propionic acid concentrations above the inhibitory value of 900 mg/L^55^ indicated in the literature were not observed (Table 3), it is known that elevated levels of propionic acid indicate H_2_ consumption.^56^ Although propionic acid can inhibit anaerobic digestion, this inhibition is often reversible, depending on system conditions, such as microbial acclimation to high concentrations of propionic acid, the shift to hydrogenotrophic methanogenesis, the presence of recalcitrant materials that help maintain pH, and the composition of the digestion mixture, which may include food waste and dairy manure, favoring the degradation of the acid.^55^

### 3.3 Influence of OLR on the Performance of Methanogenic Reactors

In this study, the OLR in the UASB-SS and UASB-TS reactors was incrementally increased, ranging from 1.5 to 22.0 g of COD/L_reactor_·day. As shown in Figure 3, a positive correlation was observed between volumetric biogas and CH_4_ production and the increasing OLR over 400 days of operation. In contrast, Cavalcante et al.^10^ reported a limiting OLR of 10.0 g COD/L_reactor_·day for a single-stage UASB treating FVW, beyond which reactor failure occurred due to VFA accumulation. These findings support the hypothesis that CH_4_ production can be optimized by employing alternative pretreatment methods and implementing TS anaerobic digestion.

**Figure 3:**
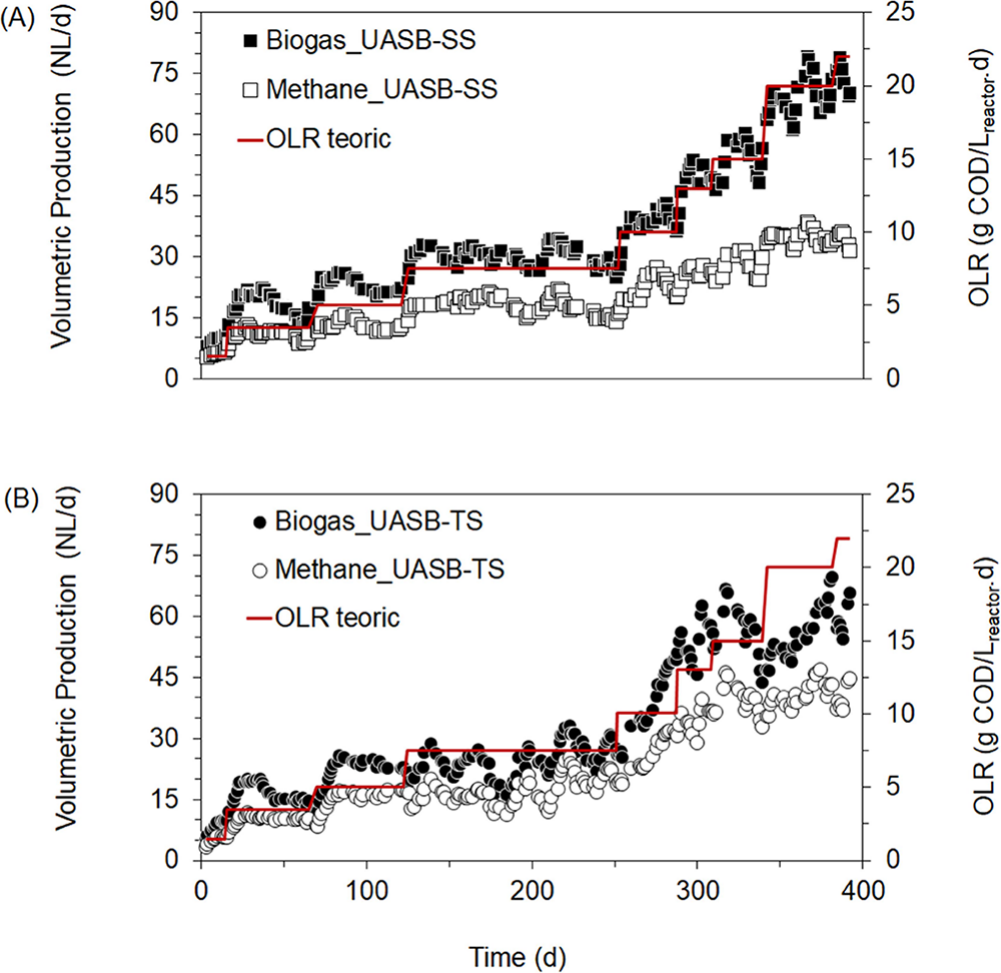
Volumetric production of biogas and CH_4_ during the operation of the methanogenic reactors (A) UASB-SS and (B) UASB-TS.

It can be inferred that the CH_4_ proportion in the UASB-SS reactor tends to decrease with the increase at the OLR, as the volumetric biogas production increased more sharply than CH_4_ production, especially during the last 150 days of operation (Figure 3A). This behavior was not observed in the UASB-TS reactor, which, despite variations in the volumetric gas production, maintained a higher CH_4_ proportion until the end of the operation (Figure 3B).

At an OLR of 20.0 g of COD/L_reactor_·day, the UASB-TS reactor achieved the highest CH_4_ percentage with 72% of the biogas, while the UASB-SS reactor showed 49%. Although the graphical observations suggest the advantage of using a TS reactor system, it is important to note that there were no indications of overloading or operational failures.

Figure 4 shows the behavior of the methanogenic reactors concerning the effluent pH and the alkalinity/partial alkalinity (IA:AP) ratio. It is important to highlight that although each operating condition was maintained until the variation in organic matter removal efficiency and methane production yield was less than 10%, the standard deviation shown in Figure 4 represents the entire operational period for each condition. As the OLR increases, reactor instability is expected, leading to a rise in the IA:PA ratio. Consequently, the IA:PA values recorded at the beginning of each operating condition contribute to higher standard deviation values. However, the standard deviation in the UASB-SS reactor was greater than in the UASB-TS reactor, indicating either higher instability in SS or a longer time required for the reactor to reach a stable IA:PA ratio.

**Figure 4:**
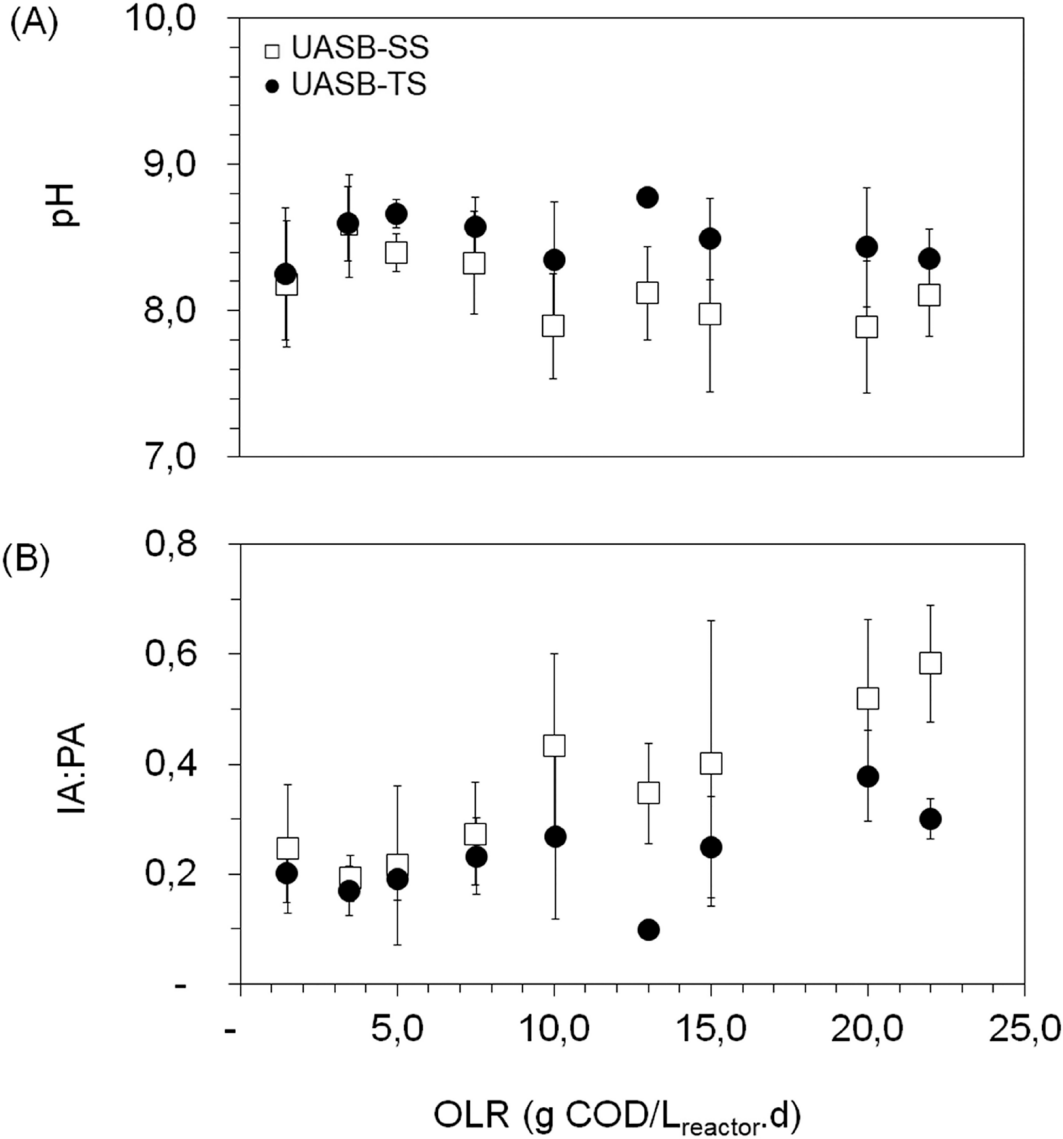
Evolution of (A) pH and (B) IA:PA ratio for the methanogenic UASB-SS and UASB-TS reactors under different Organic Loading Rates (OLR).

In general, the OLR of 10.0 g of COD/L_reactor_·day tended to be the critical point from which the UASB-TS reactor outperformed the UASB-SS reactor in all analyzed parameters. For the UASB-SS reactor used in this study, instability was observed in the OLR of 20.0 g of COD/L_reactor_·day, from which the intermediate IA:PA ratio remained above 0.5. In a recent study, Cavalcante et al.^10^ reported that, under an OLR of 10.0 g COD/L_reactor_·day, the SS UASB reactor used for the digestion of FVWL, sourced from the same CEASA as this investigation, collapsed due to VFA accumulation, presenting an IA:PA ratio >0.5, indicating a possible overload. In this work, the FVWL contained 1.2 g COD/g VS_residue_, while in the study of Cavalcante et al.,^10^ the value was 1.7 g COD/g VS_residue_. This less concentrated residue may have contributed to a greater resilience to load increases. Similarly, Tanguay-Rioux et al.^57^ reported stable performance of a UASB under an OLR of 12.0 g COD/L_reactor_·day, treating an FVW effluent with 1.3 g COD/g VS_residue_, supporting the hypothesis that the concentration of the effluent may influence reactor stability under high loads.

The UASB-TS reactor maintained an IA:PA ratio <0.4 under all applied OLRs. The pH of both reactors was controlled by the addition of an external alkalinity source (NaHCO_3_) in the influent, with averages of 8.1 ± 0.13 for the UASB-SS and 8.5 ± 0.05 for the UASB-TS. However, at an OLR of 20.0 g of COD/L_reactor_·day, the alkalizing ratio for the UASB-SS was 1.0 g of NaHCO_3_/g of COD_added_, while for the UASB-TS, it was 0.30 g of NaHCO_3_/g of COD_added_. Although a pH above 8.0 is not ideal, several studies show that UASB reactors can sustain methanogenic activity at this level.^58–60^

Zhao et al.^61^ reported that a pH of 8.0 during the acidogenic stage of food waste digestion resulted in the highest cumulative MY and the best overall energy recovery efficiency. The authors demonstrated that maintaining the pH at 8.0 allowed for a significant increase in CH_4_ production during the methanogenic phase, resulting in a MY of 412.6 mL/g VS_added_, with an energy recovery efficiency of 76.4%. Therefore, these studies suggest that although methanogenesis is possible at pH levels above 8.0, optimal performance may vary depending on the specific reactor configurations and operational conditions. However, pH levels higher than 8.0 can affect microbial communities and lead to CH_4_ production inhibition due to free ammonia nitrogen.^62^ Figure 5 presents the average data and their respective standard deviations for COD, efficiency, MY, and Methane Volumetric Production (MVP) for the ten increases in OLR applied to the two methanogenic reactors operated in parallel.

**Figure 5:**
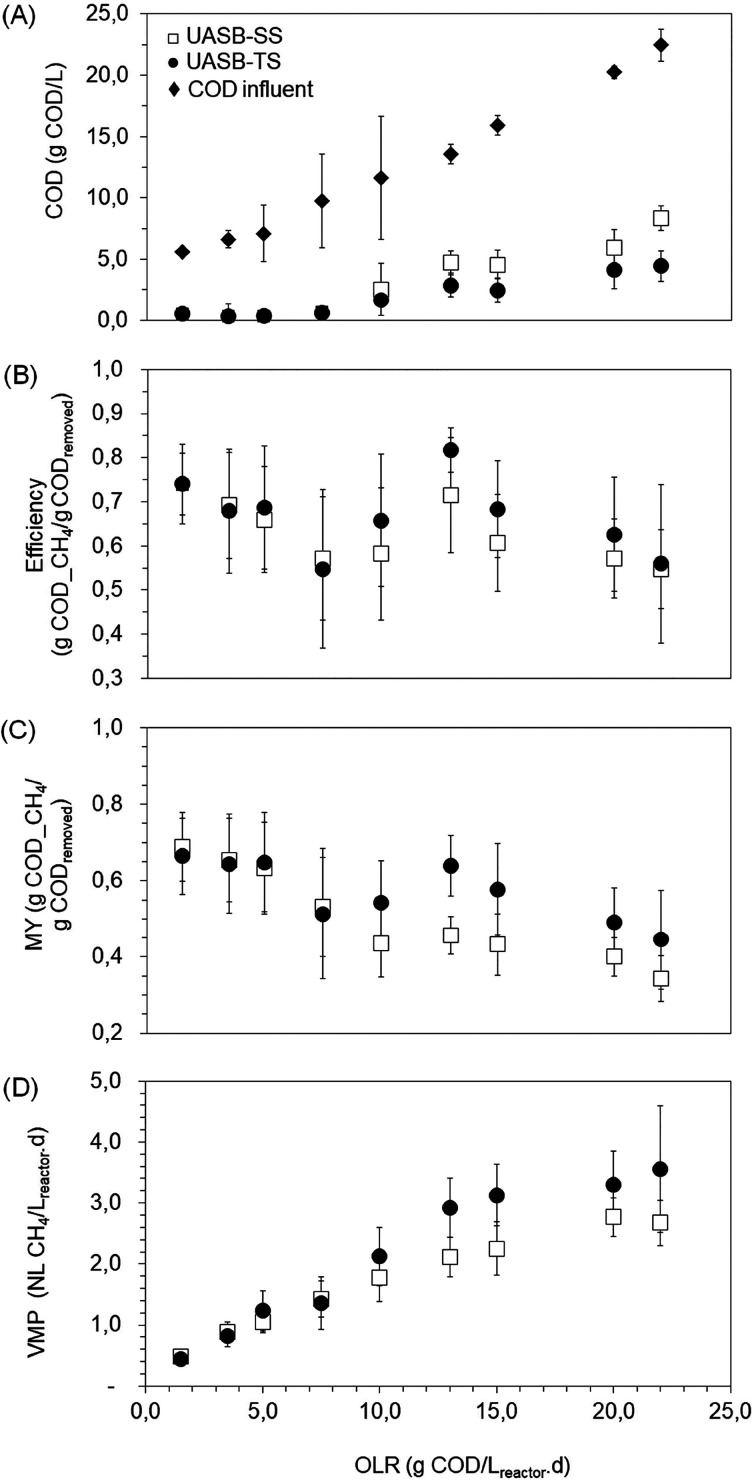
Evolution of (A) COD, (B) efficiency, (C) MY, and (D) volumetric methane production (VMP) for both methanogenic UASB-SS and UASB-TS reactors under different organic loading rates (OLR).

The OLR influences the COD removal and CH_4_ production in UASB reactors. After an OLR of 10.0 g COD/L_reactor_·day (Figure 5A), there was a significant difference (*p* < 0.05) between the COD removal averages for the UASB-SS and UASB-TS reactors, with both reactors tending to decrease their removal efficiencies as the OLR increased. A similar behavior was reported by Tanguay-rioux et al.,^57^ who reported that the UASB digester exhibited stable performance up to an OLR of 44 g COD/L_reactor_·day using the FVWL. However, both COD conversion and MY significantly declined when the OLR exceeded 10–12 g COD/L_reactor_·day. Regardless of reactor type, Almeida et al.^17^ observed that, in a SS CSTR digesting FVW, COD removal dropped from 82.2 to 25.2% as the OLR increased from 1.0 to 2.0 g COD/L_reactor_·day, a load ten times lower than that applied to the reactors in the present study. Therefore, the reactor in this study results in a smaller reactor and higher methane productivity. These findings show that high OLR can compromise the efficiency of the anaerobic digestion process, negatively impacting CH_4_ production.

At the maximum OLR applied to the reactors, the UASB-SS reactor showed a COD removal of 62.7%, while UASB-TS maintained its removal at 76.8%. Despite the difference, these are acceptable values for UASB reactors.^57,63^ However, they were considerably lower when compared to the results of Ganesh et al.^64^ These authors reported a COD removal of 83% for a SS system and 97.5% for a TS system in the anaerobic digestion of FVW, where the substrate had a COD:VS_residue_ ratio of 1.16. In comparison, the COD:VS_residue_ ratio of 1.24 in this study suggests a more complex substrate, which may explain the higher COD removal efficiency observed by Ganesh et al.^64^

Following these results, the highest COD-to-CH_4_ conversion occurred at an OLR of 12.0 g COD/L_reactor_·day for both reactors, with the UASB-TS reactor being more efficient (*p* < 0.05), achieving 0.8 g COD_CH_4_/g COD_removed_ (∼0.29 NL CH_4_/g COD_removed_) compared to 0.72 g COD_CH_4_/g COD_removed_ in the UASB-SS (Figure 5B). It is noteworthy that at this point, the pH of the UASB-TS was 8.7 with an IA:PA ratio of 0.1. From this OLR onward, the average efficiencies of both reactors showed no significant difference (*p* > 0.05).

The same behavior was observed regarding MY (Figure 5C). At an OLR of 12.0 g of COD/L_reactor_·day, the UASB-TS reactor outperformed the UASB-SS by 39%, achieving an MY of 0.64 g of COD_CH_4_/g of COD_removed_ (∼0.23 NL CH_4_/g of COD_removed_). Despite this good result, it was still lower than the 0.25 NL CH_4_/g COD_removed_ reported by Cavalcante et al.^10^ at an OLR of 10.0 g COD/L_reactor_·day in a SS UASB reactor. Similarly, Tanguay-rioux et al.^57^ achieved even higher MY (0.27 L CH_4_/g COD_added_) using the FVWL in a SS UASB reactor. One hypothesis is that the lipid nature of FVWL favors more effective degradation in a SS system, as observed by Wu et al.^65,66^ The UASB-SS reactor reached its highest VMP (Figure 5D) at an OLR of 20.0 g of COD/L_reactor_·day, from which the reactor tended to acidify, as even with the addition of 1.0 g of NaHCO_3_/g of COD_added_, the IA:AP ratio exceeded 0.5. Nevertheless, the CH_4_ productivity in the UASB-TS reactor reached its maximum of 3.58 NL CH_4_/L_reactor_·day at an OLR of 22.0 g of COD/L_reactor_·day, and even at this point, the reactor showed no instability regarding acid accumulation.

Finally, the superiority of the two-stage system was confirmed, as it demonstrated a 32% higher CH_4_ production compared to the single-stage system, even under a high OLR (22.0 g of COD/L_reactor_·day). Furthermore, the stability of pH highlights its robustness against the accumulation of VFA, ensuring greater operational reliability. Although both systems exhibited similar behaviors, the advantage of the two-stage system can be validated through a comparative analysis of the energy potential between them.

### 3.4 Energy Potential

The Lower Heating Value (LHV) at 25 °C and 1.0 atm was adopted, with H_2_ having a mass-based value of 119.95 KJ/kg and CH_4_ at 50.029 KJ/kg (NBR 15,213).^67^ The maximum VHP was 2.2 L of H_2_/L_reactor_·day at an OLR of 60 g of COD/L_reactor_·day. Regarding CH_4_, the maximum volumetric production in the UASB-TS reactor was 3.6 NL CH_4_/L_reactor_·day at an OLR of 22.0 g of COD/L_reactor_·day, while the maximum in the UASB-SS reactor was 2.8 NL CH_4_/L_reactor_·day at an OLR of 20.0 g of COD/L_reactor_/day. Therefore, the TS system presented the highest energy values, producing 75.9 KJ/day^–1^ in the AnStBR and 1479.1 KJ/day in the UASB-TS, for a total of 1554.9 KJ/day. This configuration was 35.7% more efficient than the UASB-SS, which produced 1145.8 KJ/day. Comparing only the two methanogenic reactors, it can be inferred that, at the maximum OLR applied to both (22.0 g of COD/L_reactor_·day), the UASB-TS generated 33.4% more energy than the UASB-SS, highlighting the advantage of separating the acidogenic and methanogenic stages into distinct reactors. In terms of energy produced per mass of COD added, the acidogenic reactor generated a maximum of 280.8 J/g COD_added_ under a load of 60.0 g COD/L_reactor_·day and 5,273 J/g COD_added_ in the UASB-TS under a maximum OLR of 22.0 g COD/L_reactor_·day. Meanwhile, the UASB-SS produced 3954.49 J/g COD_added_ at the same organic loading rate. In both approaches, the low energy yield of H_2_ from FVWL compared to energy produced from CH_4_ is evident; thus, in a TS system, the total energy is primarily derived from the methanogenic reactor, driven by the metabolites made available in the acidogenic reactor. This observation is consistent with other studies in the literature, indicating that the MY is substantially higher than H_2_ in TS systems. Viana et al.^68^ demonstrated that using glycerol as a substrate resulted in a maximum MY of 0.39 L CH_4_/g COD_added_, while H_2_ production was significantly lower, at only 2.41 mL H_2_/g COD_added_. Similarly, Meier^69^ when evaluating the anaerobic digestion of cassava wastewater with the addition of residual glycerol obtained 1762.1 mL CH_4_ per liter of treated waste, in contrast to 861.4 mL H_2_. Kvesitadze et al.^70^ corroborated these results, reporting that CH_4_ production (0.52 L/g VS) was much higher than H_2_ production (0.10 L/g VS) in a similar system. Therefore, it is expected that, in TS anaerobic digestion systems, CH_4_ is the primary product which, in terms of volume, provides a greater amount of energy, compensating for its lower heating value per unit mass compared to H_2_.

## 4 Conclusions

This study demonstrated that the TS anaerobic digestion system (AnStBR + UASB-TS) provided significant advantages over the UASB-SS configuration for the treatment of FVWL. The TS system achieved 20.4% higher CH_4_ production and was 35.7% more energy efficient compared with the SS system. The enhanced performance of the TS process can be attributed to improved hydrolysis in the acidogenic reactor, which ensured more readily available VFA for the methanogenic phase, resulting in stable operation even under higher OLRs. The energy analysis highlighted that the TS system was able to generate up to 1554.9 KJ/day, significantly outperforming the UASB-SS, which produced 1145.8 KJ/day. This demonstrates the superior energy recovery potential of the TS process, especially under high OLR conditions. Moreover, the ability of the TS system to maintain a stable pH and IA:PA ratio under these conditions further reinforces its robustness and operational reliability. These findings suggest that the TS anaerobic digestion system is a promising solution for improving the efficiency and sustainability of organic waste management, particularly in the context of energy recovery.

## References

[1] Kumar, D. ; Choudhuri, S. ; Shandilya, A. K. ; Singh, R. ; Tyagi, P. ; Singh, A. K. Food Waste & Sustainability Through A Lens of Bibliometric Review: A Step Towards Achieving SDG 2030. In ICISTSD 2022 - 3rd International Conference on Innovations in Science and Technology for Sustainable Development; IEEE, 2022; pp 185–192.

[2] Kaza, S. ; Yao, L. ; Bhada-Tata, P. ; Van Woerden, F. What a Waste 2.0: A Global Snapshot of Solid Waste Management to 2050; World Bank: Washington, DC, 2018.

[3] Flanagan, K. ; Robertson, K. A. I. ; Hanson, C. REDUCING FOOD LOSS AND WASTE; World Resources Institute, 2019.

[4] Durán-Sandoval D., Durán-Romero G., Uleri F. (2023). How Much Food Loss and Waste Do Countries
with Problems with Food Security Generate ?. Agriculture.

[5] FAO. Global Food Losses and Food Waste–Extent, Causes and Prevention; FAO: Rome, 2011.

[6] Kothari R., Tyagi V. V., Pathak A. (2010). Waste-to-Energy: A Way from Renewable
Energy Sources to Sustainable Development. Renewable
and Sustainable Energy Reviews.

[7] Bouallagui H., Touhami Y., Ben Cheikh R., Hamdi M. (2005). Bioreactor Performance
in Anaerobic Digestion of Fruit and Vegetable Wastes. Process Biochemistry.

[8] Zhou M., Yan B., Wong J. W. C., Zhang Y. (2018). Enhanced Volatile Fatty Acids Production
from Anaerobic Fermentation of Food Waste: A Mini-Review Focusing
on Acidogenic Metabolic Pathways. Bioresour.
Technol..

[9] Liakou V., Pateraki C., Palaiogeorgou A., Kopsahelis N., Machado A., Castro D., Maria D., Freire G., Nychas G. E., Papanikolaou S., Koutinas A. (2018). Food and Bioproducts
Processing Valorisation of Fruit and Vegetable Waste from Open Markets
for the Production of 2, 3-Butanediol. Food
Bioprod. Process..

[10] Cavalcante W. A., Aparecida C., Menezes D., Francisco C. G., Júnior S., Zaiat M., Gehring T. A., Leit R. C. (2023). From Start-up
to Maximum Loading: An Approach for Methane Production in Upflow Anaerobic
Sludge Blanket Reactor Fed with the Liquid Fraction of Fruit and Vegetable
Waste. J. Environ. Manage..

[11] Merino D., Quilez-molina A. I., Perotto G., Bassani A., Athanassiou A. (2022). A Second Life
for Fruit and Vegetable Waste: A Review on Bioplastic Films and Coatings
for Potential Food Protection Applications. Green Chem..

[12] Ferguson R. M. W., Coulon F., Villa R. (2016). Organic Loading
Rate: A Promising
Microbial Management Tool in Anaerobic Digestion. Water Res..

[13] Parra-Orobio B. A., Cruz-Bournazou M. N., Torres-Lozada P. (2021). Single-Stage and Two-Stage Anaerobic
Digestion of Food Waste: Effect of the Organic Loading Rate on the
Methane Production and Volatile Fatty Acids. Water, Air, Soil Pollut..

[14] Sebola M., Tesfagiorgis H., Muzenda E. (2015). Effect of Particle
Size on Anaerobic
Digestion of Different Feedstocks. South African
J. Chem. Eng..

[15] Agrawal A., Kumar P., Prabir C. (2023). Hydrolysis and Acidogenesis Study
of Fruit and Vegetable Waste Using Activated Sludge. Biomass Convers. Biorefin..

[16] Schievano A., Tenca A., Scaglia B., Merlino G., Rizzi A., Daffonchio D., Oberti R., Adani F. (2012). Two-Stage vs Single-Stage
Thermophilic Anaerobic Digestion: Comparison of Energy Production
and Biodegradation Efficiencies. Environ. Sci.
Technol..

[17] Almeida P. d. S., de Menezes C. A., Cavalcante W. d. A., Pinheiro N. F., da Silva Junior F. d.
C. G., Viana M. B., Leitão R. C. (2024). Mesophilic Anaerobic Digestion of Fruit and Vegetable
Waste: Single- Versus Two-Stage Reactor, and Modeling of the Liquid
and Solid Fractions of the Residue. Ind. Biotechnol..

[18] Dangol, S. ; Ghimire, A. ; Tuladhar, S. ; Khadka, A. ; Thapa, B. ; Sapkota, L. Biohythane and Organic Acid Production from Food Waste by Two-Stage Anaerobic Digestion: A Review within Biorefinery Framework; Springer: Berlin Heidelberg, 2022; vol 19.

[19] de
Menezes C. A., Duarte M. S., Teixeira I. N., Cavalcante W. d. A., Almeida P. d. S., Viana M. B., Zaiat M., Leitão R. C. (2024). Using Fruit and Vegetable Waste to Generate Hydrogen
through Dark Fermentation. Engenharia Sanitaria
e Ambiental.

[20] de
Menezes C. A., Santos D. R. d., Cavalcante W. d. A., Almeida P. d. S., Silva T. P., Júnior F. d. C. G. d. S., Gehring T. A., Zaiat M., Santos A. B. d., Leitão R. C. (2024). Innovative System to Maximize Methane
Production from Fruit and Vegetable Waste. Environ.
Sci. Pollut. Res..

[21] APHA; AWWA; WEF . Standard Methods for the Examination of Water and Wastewater, 22nd edition; Rice, E. W. ; Baird, R. B. ; Eaton, A. D. ; Clesceri, L. S. , Eds.; Elsevier: Washington, DC, 2012.

[22] Dubois M., Gilles K. A., Hamilton J. K., Rebers P. A., Smith F. (1956). Colorimetric
Method for Determination of Sugars and Related Substances. Anal. Chem..

[23] Maintinguer S. I., Fernandes B. S., Duarte I. C. S., Saavedra N. K., Adorno M. A. T., Varesche M. B. (2008). Fermentative Hydrogen Production by Microbial Consortium. Int. J. Hydrogen Energy.

[24] Mockaitis G., Rodrigues A. D., Zaiat M., Ratusznei S. M., Foresti E. (2006). Anaerobic Whey Treatment
by a Stirred Sequencing Batch
Reactor (ASBR): Effects of Organic Loading and Supplemented Alkalinity. Journal of environmental Managment.

[25] Ripley L. E., Boyle W. C., Converse J. C. (1986). Improved
Alkalimetric Monitoring
for Anaerobic Digestion of High-Strength Wastes. Water Pollut. Control Federation.

[26] DiLallo R., Albertson O. E. (1961). Volatile
Acids By Direct Titration. Water Pollut. Control.

[27] Sitorus B., Sukandar, Panjaitan S. D. (2013). Biogas
Recovery from Anaerobic Digestion Process of Mixed Fruit -Vegetable
Wastes. Energy Procedia.

[28] Martínez-Mendoza L. J., Lebrero R., Muñoz R., García-Depraect O. (2022). Influence
of Key Operational Parameters on Biohydrogen Production from Fruit
and Vegetable Waste via Lactate-Driven Dark Fermentation. Bioresour. Technol..

[29] Ambaye T. G., Rene E. R., Dupont C., Wongrod S., van Hullebusch E. D. (2020). Anaerobic
Digestion of Fruit Waste Mixed With Sewage Sludge Digestate Biochar:
Influence on Biomethane Production. Frontiers
in Energy Research.

[30] Dwivedi A. H., Gedam V. V., Kumar M. S. (2020). Sustainable
Hydrogen Production from
Fruit and Vegetable Waste (FVW) Using Mixed Anaerobic Cultures via
Dark Fermentation: Kinetic Aspects. International
Journal of Energy and Environmental Engineering.

[31] Agrawal A., Chaudhari P., Ghosh P. (2023). Anaerobic Digestion of Fruit and
Vegetable Waste: A Critical Review of Associated Challenges. Environmental Science and Pollution Research.

[32] Speece, R. E. Environmental Requirements for Anaerobic Digestion of Biomass; Springer, 1985; vol 2, p 51.

[33] Bryant, M. P. ; Tzeng, S. F. ; Robinson, I. M., Jr. ; Nutrient Requirements of Methanogenic Bacteria. In Anaerobic Biological Treatment Processes; Advances in Chemistry; American Chemical Society: Washington, DC, 1971; pp 23–40.

[34] Jiang Y., Heaven S., Banks C. J. (2012). Strategies
for Stable Anaerobic Digestion
of Vegetable Waste. Renewable Energy.

[35] Sawalha H., Maghalseh M., Qutaina J., Junaidi K., Rene R. (2020). Removal of
Hydrogen Sulfide from Biogas Using Activated Carbon Synthesized from
Different Locally Available Biomass Wastes - a Case Study from Palestine. Bioengineered.

[36] Angelidaki I., Alves M., Bolzonella D., Borzacconi L., Campos J. L., Guwy A. J., Kalyuzhnyi S., Jenicek P., Van Lier J. B. (2009). Defining the Biomethane Potential
(BMP) of Solid Organic Wastes and Energy Crops: A Proposed Protocol
for Batch Assays. Water Sci. Technol..

[37] Tang J., Wang X. C., Hu Y., Zhang Y., Li Y. (2017). Effect of
PH on Lactic Acid Production from Acidogenic Fermentation of Food
Waste with Different Types of Inocula. Bioresour.
Technol..

[38] Balmant W., Oliveira B. H., Mitchell D. A., Vargas J. V. C., Ordonez J. C. (2014). Optimal
Operating Conditions for Maximum Biogas Production in Anaerobic Bioreactors. Applied Thermal Engineering.

[39] García-Depraect O., Castro-Muñoz R., Muñoz R., Rene E. R., León-Becerril E., Valdez-Vazquez I., Kumar G., Reyes-Alvarado L. C., Martínez-Mendoza L. J., Carrillo-Reyes J., Buitrón G. (2021). A Review on the Factors Influencing Biohydrogen Production
from Lactate: The Key to Unlocking Enhanced Dark Fermentative Processes. Bioresour. Technol..

[40] Ordoñez-Frías E. J., Muñoz-Páez K. M., Buitrón G. (2024). Biohydrogen
Production from Fermented Acidic Cheese Whey Using Lactate: Reactor
Performance and Microbial Ecology Analysis. Int. J. Hydrogen Energy.

[41] Brodowski F., Łężyk M., Gutowska N., Oleskowicz-Popiel P. (2022). Effect of
External Acetate on Lactate-Based Carboxylate Platform: Shifted Lactate
Overloading Limit and Hydrogen Co-Production. Sci. Total Environ..

[42] Martínez-Mendoza L. J., Lebrero R., Muñoz R., Garíca-Depraect O. (2022). Influence
of Key Operational Parameters on Biohydrogen Production from Fruit
and Vegetable Waste via Lactate-Driven Dark Fermentation. Bioresour. Technol..

[43] Martínez-mendoza L. J., García-depraect O., Mu R. (2023). Unlocking the High-Rate
Continuous Performance of Fermentative Hydrogen Bioproduction from
Fruit and Vegetable Residues by Modulating Hydraulic Retention Time. Bioresour. Technol..

[44] Wainaina S., Awasthi M. K., Horváth I. S., Taherzadeh M. J. (2020). Anaerobic
Digestion of Food Waste to Volatile Fatty Acids and Hydrogen at High
Organic Loading Rates in Immersed Membrane Bioreactors. Renewable Energy.

[45] Zhang S., Lee Y., Kim T. H., Hwang S. J. (2013). Effects
of OLRs and HRTs on Hydrogen
Production from High Salinity Substrate by Halophilic Hydrogen Producing
Bacterium (HHPB). Bioresour. Technol..

[46] Paudel S., Kang Y., Yoo Y. S., Seo G. T. (2017). Effect of Volumetric
Organic Loading Rate (OLR) on H2 and CH4 Production by Two-Stage Anaerobic
Co-Digestion of Food Waste and Brown Water. Waste Management.

[47] Araujo M. N., Fuess L. T., Cavalcante W. A., Couto P. T., Rogeri R. C., Adorno M. A. T., Sakamoto I. K., Zaiat M. (2024). Fixed Bed in Dark Fermentative
Reactors: Is It Imperative for Enhanced Biomass Retention, Biohydrogen
Evolution and Substrate Conversion?. Int. J.
Hydrogen Energy.

[48] Jain R., Panwar N. L., Jain S. K., Gupta T., Agarwal C., Meena S. S. (2024). Bio-Hydrogen Production
through Dark Fermentation:
An Overview. Biomass Conversion and Biorefinery.

[49] Noguer M. C., Magdalena J. A., Bernet N., Escudié R., Trably E. (2022). Enhanced Fermentative Hydrogen Production from Food
Waste in Continuous Reactor after Butyric Acid Treatment. Energies.

[50] Nicolaou S. A., Gaida S. M., Papoutsakis E. T. (2010). A Comparative
View of Metabolite
and Substrate Stress and Tolerance in Microbial Bioprocessing: From
Biofuels and Chemicals, to Biocatalysis and Bioremediation. Metab Eng..

[51] Zhu Y., Yang S. T. (2004). Effect of PH on Metabolic Pathway Shift in Fermentation
of Xylose by Clostridium Tyrobutyricum. J. Biotechnol..

[52] Khanal S. K., Chen W. H., Li L., Sung S. (2004). Biological Hydrogen
Production: Effects of PH and Intermediate Products. Int. J. Hydrogen Energy.

[53] Moon C., Jang S., Yun Y. M., Lee M. K., Kim D. H., Kang W. S., Kwak S. S., Kim M. S. (2015). Effect of the Accuracy
of PH Control on Hydrogen Fermentation. Bioresour.
Technol..

[54] van
Ginkel S., Logan B. E. (2005). Inhibition of Biohydrogen Production
by Undissociated Acetic and Butyric Acids. Environ.
Sci. Technol..

[55] Han Y., Green H., Tao W. (2020). Reversibility of Propionic Acid Inhibition
to Anaerobic Digestion: Inhibition Kinetics and Microbial Mechanism. Chemosphere.

[56] Wang B., Wan W., Wang J. (2008). Inhibitory
Effect of Ethanol, Acetic Acid, Propionic
Acid and Butyric Acid on Fermentative Hydrogen Production. Int. J. Hydrogen Energy.

[57] Tanguay-rioux F., Spreutels L., Roy C., Frigon J. (2024). Assessment of the Feasibility
of Converting the Liquid Fraction Separated from Fruit and Vegetable
Waste in a UASB Digester. Bioengineering.

[58] Mischopoulou M., Kalamaras S. D., Naidis P., Kotsopoulos T. A., Samaras P. (2017). Start-up and Steady-State
Results of a UASB Reactor
Treating High PH Baker’s Yeast Molasses Wastewater for Methane
Production. J. Chem. Technol. Biotechnol..

[59] Gomes N. A., Almeida M. V. d. A., de Melo M. C., Monteiro V. E. D., de
Oliveira R. (2018). Influence of Physical-Chemical Parameters on the Composition
of Toxic Constituents in Landfill Leachate. Rev. Mater..

[60] Ajayi-Banji A., Rahman S. (2022). A Review of Process
Parameters Influence in Solid-State
Anaerobic Digestion: Focus on Performance Stability Thresholds. Renewable and Sustainable Energy Reviews.

[61] Zhao Q., Arhin S. G., Yang Z., Liu H., Li Z., Anwar N., Papadakis V. G., Liu G., Wang W. (2021). PH Regulation
of the First Phase Could Enhance the Energy Recovery from Two-Phase
Anaerobic Digestion of Food Waste. Water Environment
Research.

[62] Capson-Tojo G., Moscoviz R., Astals S., Robles, Steyer J. P. (2020). Unraveling the Literature
Chaos around Free Ammonia Inhibition in Anaerobic Digestion. Renewable Sustainable Energy Rev..

[63] Callado N. H., Helena M., Damianovic Z., Foresti E. (2017). Influência da
Razão DQO /[ SO 42- ] e da Concentração de Na
+ na Remoção de Matéria Orgânica e Sulfato
Em Reator UASB. Eng. Sanit. Ambient..

[64] Ganesh R., Torrijos M., Sousbie P., Lugardon A., Steyer J. P., Delgenes J. P. (2014). Single-Phase and
Two-Phase Anaerobic Digestion of Fruit
and Vegetable Waste: Comparison of Start-up, Reactor Stability and
Process Performance. Waste Management.

[65] Wu L. J., Kobayashi T., Li Y. Y., Xu K. Q. (2015). Comparison of Single-Stage
and Temperature-Phased Two-Stage Anaerobic Digestion of Oily Food
Waste. Energy Conversion and Management.

[66] Wu Y., Ma H., Zheng M., Wang K. (2015). Lactic Acid Production from Acidogenic
Fermentation of Fruit and Vegetable Wastes. Bioresour. Technol..

[67] ABNT. NBR 15213 - Calculation of Calorifc Values, Density, Relative Density and Wobbe Index of Combustible Gases from Composition; ABNT, 2008; p 45.

[68] Viana M. B., Augusto E., Vasconcelos F., de Menezes C. A., Almeida P. D. S., Sandra T. S., Leitão R. C. (2024). Hydrogen
and Methane Production in Two-Stage Upflow Anaerobic Sludge Blanket
Reactors from Crude Glycerol. Ind. Biotechnol..

[69] Meier, T. R. W. Produção de Hidrogênio e Metano a Partir de Manipueira com Adição de Glicerol Residual por Biodigestão Anaeróbia; State University of Western Paraná, 2020.

[70] Kvesitadze G., Sadunishvili T., Dudauri T., Zakariashvili N., Partskhaladze G., Ugrekhelidze V., Tsiklauri G., Metreveli B., Jobava M. (2011). Two-Stage Anaerobic Process for Bio-Hydrogen
and Bio-Methane Combined Production from Biodegradable Solid Wastes. Energy.

